# A new technique to ATTACK the silent pandemic of antimicrobial resistance

**DOI:** 10.1002/mlf2.12065

**Published:** 2023-05-17

**Authors:** Yong‐Guan Zhu

**Affiliations:** ^1^ Key Laboratory of Urban Environment and Health, Institute of Urban Environment Chinese Academy of Sciences Xiamen China; ^2^ State Key Laboratory of Urban and Regional Ecology, Research Centre for Eco‐environmental Sciences Chinese Academy of Sciences Beijing China

Due to the global proliferation of antibiotic‐resistant bacteria (ARB) and antibiotic resistance genes (ARGs) in humans, animals, and the environment, antibiotic resistance has become a silent pandemic threatening public health across the globe^1^. Anthropogenic activities, including clinical antibiotic use, intensive animal farming, and landfill waste, have been identified as the greatest risk factors for the dissemination of antibiotic resistance^2^, ^3^. Additionally, the rise in disinfectant use during the COVID‐19 pandemic has exacerbated the situation by facilitating the spread of ARB^4^. Therefore, it is imperative to develop safe and effective strategies to combat this silent pandemic. These strategies must focus on reducing the presence of ARB and ARGs in the environment while also accelerating the implementation of the “ONE EARTH, ONE HEALTH” action plan.

The CRISPR‐Cas immune system found in prokaryotes has been developed into precise antimicrobials capable of selectively eradicating ARB or ARGs within complex bacterial communities^5^, ^6^. However, these antimicrobials are not foolproof, as bacteria can develop resistance to them through various mechanisms such as the inactivation of Cas nucleases with anti‐CRISPR proteins or breaking down their encoding genes via genomic rearrangements or transposition events^7^, ^8^. This results in the failure of ARB eradication or ARG reduction.

A recent study published in *Nature Communications* by Wang et al.^9^ introduced an advanced antimicrobial strategy that combines CRISPR‐Cas with the newly discovered Type VIII toxin‐antitoxin CreTA^10^, which, in prokaryotic genomes, tightly associates with CRISPR‐Cas and protects it from inactivation. If the CRISPR‐Cas system remains active after the antimicrobial being delivered into the antibiotic‐resistant (AR) pathogens, DNA breakage will occur, resulting in the death of the pathogen or its increased sensitivity to antibiotics upon the loss or mutation of target AR genes. In the event of the pathogens deactivating CRISPR‐Cas through various anti‐CRISPR mechanisms, CreTA acts as a backup system by inducing cellular death, creating a dilemma for the target pathogen^9^ (Figure 1). The authors named this innovative approach “ATTACK” (AssociaTe Toxin‐Antitoxin and CRISPR‐Cas to Kill Pathogens). The study has demonstrated that ATTACK outperforms the use of CRISPR alone by showing higher efficacy in eliminating targeted AR pathogens (in cases where the targeted ARG is located on the chromosome) and a more comprehensive reduction of ARG (in cases where it is situated on a plasmid)^9^.

**Figure 1:**
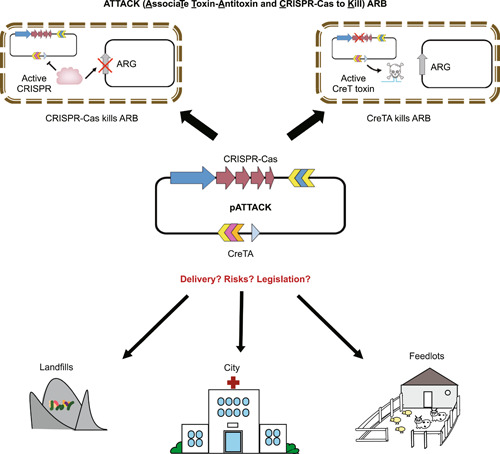
The principle, potential applications, and challenges associated with the implementation of the “ATTACK” (AssociaTe Toxin‐Antitoxin and CRISPR‐Cas to Kill Pathogens) technique. ARB, antibiotic‐resistant bacteria; ARG, antibiotic resistance gene; CreTA, CRISPR‐regulated toxin‐antitoxin.

I believe that ATTACK is an advanced and precise antimicrobial technique against ARB. While the authors have demonstrated its efficacy in targeting the carbapenem‐resistant pathogenic bacterium *Acinetobacter baumannii*, potentially, it could also prove to be a valuable tool for combating the diverse range of ARB and ARGs found in the environment. In conjunction with efficient delivery vectors that may be developed in the future, for example, engineered phages, conjugative plasmids, or bacteria‐targeting nanoparticles, ATTACK has the potential to help “attack” the silent, yet perilous pandemic of antimicrobial resistance. Moreover, it will be crucial to conduct a thorough evaluation of the potential risks associated with introducing this novel technique in the environment (Figure 1). This will require the formulation of appropriate guidelines and legal frameworks to ensure safe and responsible implementation. Given the widespread of antimicrobial resistance in medical and general environments, we must act swiftly to curtail its further dissemination. Therefore, ATTACK, when fully developed, will be instrumental in mitigating the health impacts of antimicrobial resistance, particularly under the One Health framework.

## References

[1] Larsson DGJ , Flach CF . Antibiotic resistance in the environment. Nat Rev Microbiol. 2022;20:257–69. 34737424 10.1038/s41579-021-00649-x PMC8567979

[2] Zhu YG , Johnson TA , Su JQ , Qiao M , Guo GX , Stedtfeld RD , et al. Diverse and abundant antibiotic resistance genes in Chinese swine farms. Proc Natl Acad Sci USA. 2013;110:3435–40. 23401528 10.1073/pnas.1222743110 PMC3587239

[3] Zhu YG , Zhao Y , Li B , Huang CL , Zhang SY , Yu S , et al. Continental‐scale pollution of estuaries with antibiotic resistance genes. Nat Microbiol. 2017;2:16270. 28134918 10.1038/nmicrobiol.2016.270

[4] Lu J , Guo J . Disinfection spreads antimicrobial resistance. Science. 2021;371:474. 10.1126/science.abg4380 33510019

[5] Bikard D , Euler CW , Jiang W , Nussenzweig PM , Goldberg GW , Duportet X , et al. Exploiting CRISPR‐Cas nucleases to produce sequence‐specific antimicrobials. Nat Biotechnol. 2014;32:1146–50. 25282355 10.1038/nbt.3043 PMC4317352

[6] Citorik RJ , Mimee M , Lu TK . Sequence‐specific antimicrobials using efficiently delivered RNA‐guided nucleases. Nat Biotechnol. 2014;32:1141–5. 25240928 10.1038/nbt.3011 PMC4237163

[7] Uribe RV , Rathmer C , Jahn LJ , Ellabaan MMH , Li SS , Sommer MOA . Bacterial resistance to CRISPR‐Cas antimicrobials. Sci Rep. 2021;11:17267. 34446818 10.1038/s41598-021-96735-4 PMC8390487

[8] Pawluk A , Davidson AR , Maxwell KL . Anti‐CRISPR: discovery, mechanism and function. Nat Rev Microbiol. 2018;16:12–7. 29062071 10.1038/nrmicro.2017.120

[9] Wang R , Shu X , Zhao H , Xue Q , Liu C , Wu A , et al. Associate toxin‐antitoxin with CRISPR‐Cas to kill multidrug‐resistant pathogens. Nat Commun. 2023;14:2078. 37045931 10.1038/s41467-023-37789-y PMC10097628

[10] Li M , Gong L , Cheng F , Yu H , Zhao D , Wang R , et al. Toxin‐antitoxin RNA pairs safeguard CRISPR‐Cas systems. Science. 2021;372:eabe5601. 33926924 10.1126/science.abe5601

